# The Acceptability of Adherence Support via Mobile Phones for Antituberculosis Treatment in South India: Exploratory Study

**DOI:** 10.2196/37124

**Published:** 2022-05-13

**Authors:** Nisha K Jose, Clint Vaz, Peter R Chai, Rashmi Rodrigues

**Affiliations:** 1 Non Communicable Diseases Division Indian Council of Medical Research New Delhi India; 2 Department of Emergency Medicine Brigham and Women's Hospital Boston, MA United States; 3 Department of Psychosocial Oncology and Palliative Care Dana Farber Cancer Institute Boston, MA United States; 4 Massachusetts Institute of Technology The Koch Institute for Integrated Cancer Research Cambridge, MA United States; 5 The Fenway Institute Boston, MA United States; 6 Department of Community Health St John's Medical College Bangalore India

**Keywords:** adherence, tuberculosis, antitubercular therapy, mHealth, mobile health, digital health, South India, technology acceptance, health intervention

## Abstract

**Background:**

India has the greatest burden of tuberculosis (TB). However, over 15% of the people on antitubercular therapy (ATT) in India are nonadherent. Several adherence monitoring techniques deployed in India to enhance ATT adherence have had modest effects. Increased adoption of mobile phones and other technologies pose potential solutions to measuring and intervening in ATT adherence. Several technology-based interventions around ATT adherence have been demonstrated in other countries.

**Objective:**

The objective of our study was to understand the acceptance of mobile phone adherence supports for ATT using self-administered quantitative measures among patients with TB in South India.

**Methods:**

This exploratory study was conducted at a TB treatment center (TTC) at a tertiary care center in Thrissur District, Kerala, India. We recruited 100 patients with TB on ATT using convenience sampling after obtaining written informed consent. Trained study staff administered the questionnaire in Malayalam, commonly spoken in Kerala, India. We used frequency, mean, median, and SD or IQR to describe the data.

**Results:**

Of the 100 participants diagnosed with TB on ATT, 90% used mobile phones routinely, and 84% owned a mobile phone. Ninety-five percent of participants knew how to use the calling function, while 65% of them did not know how to use the SMS function on their mobile phone. Overall, 89% of the participants did not consider mobile phone–based ATT adherence interventions an intrusion in their privacy, and 93% did not fear stigma if the adherence reminder was received by someone else. Most (95%) of the study participants preferred mobile phone reminders instead of directly observed treatment, short-course. Voice calls (n=80, 80%) were the more preferred reminder modality than SMS reminders (n=5, 5%).

**Conclusions:**

Mobile phones are likely an acceptable platform to deliver ATT adherence interventions among individuals with TB in South India. Preference of voice call reminders may inform the architecture of future adherence interventions surrounding ATT in South India.

## Introduction

Tuberculosis (TB) continues to be a significant public health problem globally, with an estimated 10 million new cases in 2019 [1]. It contributes significantly to global mortality, with an estimated 1.2 million TB-related deaths in HIV-negative individuals and 208,000 deaths among people living with HIV. Globally, India accounted for 26% of new cases of TB and carries the greatest burden of multidrug-resistant TB (MDR-TB; 27%). It also ranks first among the countries in which more than 15% of patients with TB and their households have catastrophic expenditures (>20% of total annual household income) on health in terms of total TB cases [1].

Management of TB centers around adherence to antibiotic regimens. Treatment adherence reduces the risk of developing MDR-TB while treating TB. Despite the promise of antitubercular therapy (ATT), nonadherence continues to be a significant problem due to various issues including access to medication, duration of treatment, syndemics such as AIDS and substance use disorder, and lack of routine medication taking behavior [2-6]. In India, it is estimated that >15% of patients with TB on ATT are nonadherent during their treatment regimen [7]. Given the importance of adherence in TB treatment, multiple strategies including mobile phone notifications, digital pillboxes, and ingestible sensors have been employed to ensure access to ATT, adherence to therapy, and persistence of adherence [8-11].

Since 2015, India has enacted national TB guidelines to promote mobile technologies such as 99DOTS, Video Directly Observed Therapy (vDOT), or medication event monitoring systems (MEMS) to support ATT adherence. These strategies are generally linked to mobile or smartphones where users are asked to call a phone number uncovered after opening a pill blister pack to note adherence (99DOTS) [12], use the smartphone video camera to connect or record video of ATT ingestion (vDOT), or link an adherence device to access ATT ingestion patterns (MEMS). In parallel with an emphasis on digital health technologies to measure ATT adherence in India has been a rise in mobile phone and smartphone usage. Currently there are over 1 billion mobile phone users and over 600 million internet users in India [13,14]. In contrast, a lack of familiarity with phones, unstable cellular networks outside of large metropolitan centers, and cost of phones continue to be significant barriers to mobile phone uptake in India.

Given the increasing uptake of mobile phones and their critical role in ATT adherence strategies, we undertook this pilot study in Thrissur District, Kerala, India, to explore the acceptability of adherence support for ATT delivered via mobile phones. The results of this study would help in continued research in developing an ATT adherence monitoring using mobile phones and cumulatively could help in the deployment of such a system in the community in future.

## Methods

### Recruitment

We conducted a descriptive survey study of 100 patients diagnosed with TB actively on ATT at TB treatment centers (TTCs) in a tertiary care center and other neighboring urban TB treatment centers in Thrissur. These centers manage approximately 300 patients initiated on ATT annually. Participants visiting the TTC were screened and enrolled in the study consecutively if they were over 18 years old and enrolled in the directly observed treatment, short-form (DOTS) program for at least 2 weeks. Minors and individuals who did not speak Malayalam or English were excluded. Potential participants were approached by study staff who confirmed eligibility criteria and described study procedures. Written informed consent was obtained from participants. Next, we administered a quantitative questionnaire to the participant in their local language to understand the use of mobile phones, willingness to engage with mobile phone-based ATT adherence support, and the duration and type of mobile support that would be most acceptable to participants. We also collected baseline demographics and clinical details of the participant from the TB registry at the clinic (Multimedia Appendix 1). The survey was adapted from a previous study conducted in Karnataka, India, that sought to understand the acceptance and feasibility of mobile phone–based interventions for ATT adherence [15]. The adapted survey was tested among the study team members for clarity prior to deployment.

### Ethical Considerations

Ethical approval for the study protocol and written consent was obtained from the institutional ethics committee of Amala Institute of Medical Sciences, Thrissur (AIMSEC/21/2018).

### Data Analysis

Data were entered into Microsoft Excel and analyzed using SPSS (version 23; IBM Corp). The data were described using frequencies and measures of central tendency (mean and median) and dispersion (SDs and IQRs) as appropriate.

## Results

### Overview

During the study period, we screened 115 individuals, of whom 100 met the inclusion criteria, signed consent forms, and completed all study procedures. We excluded 15 participants with TB who did not speak Malayalam or English. The mean age of our participants was 44.48 (SD 16.40) years; 69 were male, and 80 were residents of Thrissur. Five reported no formal education, while 79 of individuals reported having less than graduate training. Finally, 57 were currently unemployed. General demographics are described in Table 1.

**Table 1:** General demographics and characteristics of patients with tuberculosis (N=100).

Sociodemographic data		Value
**Sex, n**		
	Male	69
	Female	31
**Marital status, n**		
	Married	76
	Unmarried	24
**Area of residence, n**		
	Urban	20
	Rural	80
**Education level, n**		
	No formal	5
	School education^a^	79
	Graduate/university	13
	Postgraduate	3
**Employment status, n**		
	Employed	43
	Not employed	57
Age (years), mean (SD)		44.5 (16.4)
**Native language, n**		
	Malayalam	99
	Tamil	1
	Hindi	0
	English	0
	Other	0
**Reading and writing literacy, n**		
	Malayalam	86
	Tamil	3
	Hindi	2
	English	4
	Other	9
**Registration group** **(n=99), n**		
	New case^b^	86
	Relapse ^c^	9
	Default^d^	2
	Failure^e^	2
**Type of tuberculosis, n**		
	Pulmonary	64
	Extrapulmonary	36
**Sputum type, n**		
	Positive	58
	Negative	42
**HIV status, n**		
	Positive	3
	Negative	96
	Unknown	1
**Treatment phase, n**		
	Intensive	57
	Continuation	43
**Category of treatment, n**		
	Category 1^f^	87
	Category 2^g^	7
	DOTS Plus^h^	3
	Non-DOTS^i^	3
**Travelling to the DOTS center, n**		
	Yes	64
	No	36
Distance to the DOTS center (km), mean (SD)		10.8 (11.2)
Cost of travel to the DOTS center (INR; INR 1=US $0.013), mean (SD)		17.3 (26.2)
**DOTS appointment missed, n**		
	Yes	16
	No	84

^a^Any schooling less than a college education.

^b^A patient who has never had treatment for tuberculosis or has taken antituberculosis drugs for less than 1 month.

^c^A patient previously treated for tuberculosis who has been declared cured or completed in their most recent treatment episode and is presently diagnosed with bacteriologically confirmed or clinically diagnosed tuberculosis.

^d^A patient who was previously treated for TB but was lost to follow-up for 2 months or more in their most recent course of treatment and is currently diagnosed with either bacteriologically confirmed or clinically diagnosed tuberculosis.

^e^A patient who has been previously treated for tuberculosis and whose sputum smear or culture was positive at 5 months or later during treatment.

^f^New smear-positive patients with pulmonary tuberculosis.

^g^Sputum smear–positive patients who have relapsed, experienced treatment failure, or are receiving treatment after treatment interruption.

^h^DOTS Plus: DOTS + diagnosis, treatment, and management of multidrug-resistant tuberculosis.

^i^Any management other than DOTS.

### Tuberculosis Characteristics

Eighty-six of our study participants were newly diagnosed with tuberculosis within the past 6 months, and 9 had experienced recurrence after category 1 treatment. Over half (n=64) of the participants were diagnosed with pulmonary TB, of whom 58 were diagnosed with sputum-positive TB and others with sputum-negative chest-symptomatic TB. There were 3 participants with an HIV-TB coinfection. Most participants (n=57) were in the intensive phase of ATT (ie, were taking isoniazid, rifampicin, pyrazinamide, and ethambutol for 2 months), and the rest were in the continuation phase (ie, were taking isoniazid and rifampicin for 4 months) of the treatment. Sixty-four of our participants reported traveling to DOTS centers using public or personal transport and reported spending an average of INR 17.3 (US $0.2) to reach the nearest TTC for each visit. Sixteen participants reported that they had missed their DOTS appointments at least once prior to the survey. The TB characteristics are described in Table 1.

### Ownership and Basic Mobile Phone Functionality

Of the 100 participants, 90 reported routine use of mobile phones, among whom 39 did not have a camera on their phone, 37 did not know how to use the camera, and 54 did not use any phone function other than calling. Of those who used mobile phones, 84 were the primary owner of the phone and owned at least one phone for a median duration of 6 (IQR 3, 10) years (Table 2). The majority (n=95) reported competence with using voice calls, but 65 did not know how to operate the text messaging function on their phone. When asked about participants usage of mobile phones for connecting with health care facilities, 62 among reached out to their health care provider when they were unwell and 34 of them used it to know the availability of their doctors. Ownership and basic mobile phone functionality are described in Table 2.

**Table 2:** Ownership and mobile phone functionality.

Ownership and mobile phone functionality		Value
**Routine use of mobile phones (daily use), n**		
	Yes	90
	No	10
**Phone ownership, n**		
	Own a phone	84
	Own a phone but share it with other family members	1
	Shared a phone owned by another family member	5
	No phone	10
Duration of phone use (years), median (IQR)		6 (3, 10)
**Use of calling function, n**		
	Yes	95
	No	4
	Not answered	1
**Use of the SMS function in the phone, n**		
	Yes	34
	No	65
	Not answered	1
**Camera function available in the phone, n**		
	Yes	61
	No	39
**Use of camera function on phone, n**		
	Yes	63
	No	37
**Do you use the alarm function?, n**		
	Yes	49
	No	51
**Other usages of mobile phones, n**		
	Listen to the radio	16
	Play games	10
	Watch or stream videos	13
	Others	7
	None	54
**In the past, have you used a phone (mobile or landline) for any of the following?, n**		
	Calls for medical-related complaints to the health care team	62
	Scheduling physician appointments	34
	Coordinate pick up of antitubercular therapy medication	1
	Purchasing medications	2
	Others	2
	No use of phones for any health-related purposes	2

### Acceptability of Mobile Phone Interventions for ATT Adherence

Next, we asked participants about their willingness to use mobile phones to receive messages surrounding ATT adherence (Table 3). Eighty-nine participants were accepting of mobile phone ATT adherence supports, and 93 participants did not fear stigma if ATT adherence reminders were received by someone else. Seventy-four participants reported that they would prefer to use mobile phone reminders as a mode of ATT adherence monitoring, and 95 participants preferred mobile phone reminders instead of current DOTS therapy. Additionally, 78 participants reported that they would be willing to discuss their medical care and adherence with their ATT provider using their mobile phone. When asked around ideal methods for reminders, the majority (n=80) preferred telephone call reminders. When asked about the frequency of reminders, 79 participants reported that they would prefer daily reminders. Malayalam was the preferred language for telephone call (n=79) and text message (n=71) reminders, and they would like to receive it during morning hours between 6 AM and 10 AM (n=80).

**Figure 1:**
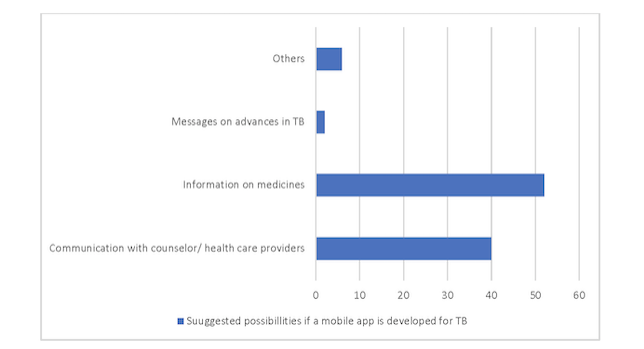
Suggested possibilities if the mobile app is developed for tuberculosis. TB: tuberculosis.

We additionally asked about participant preferences to engage with a mobile app to manage and promote ATT adherence. Forty participants indicated they would also use such an app for communication with a counselor or health care providers and 52 would seek information on their prescribed pharmacotherapy via the app (Figure 1).

**Table 3:** Acceptability of mobile phone usage for ATT^a^ adherence and preferences.

Acceptability of mobile phone usage for ATT adherence			Value, n
**Mobile phones for medication adherence as an intrusion in a person’s life**			
	Yes	9	
	No	89	
	Do not know	2	
**Fear of stigma if ATT adherence reminder received by someone else**			
	Yes	7	
	No	93	
**Preference if given a choice to select an adherence monitoring method**			
	Continue current DOTS therapy	20	
	Mobile phone reminders	74	
	Discontinue monitoring method	6	
**Interactive mobile phone reminders instead of DOTS therapy for ATT adherence**			
	Yes	95	
	No	5	
**Reminder preference for ATT adherence support**			
	Telephone call (audio format)	80	
	SMS message	15	
	No preference	5	
**Language preferred for the telephone call**			
	Malayalam	79	
	English	20	
	Others	1	
**Language preferred for SMS text messages**			
	Malayalam	71	
	English	28	
	Either English/Malayalam	1	
**Frequency of reminders**			
	As often as the medications need to be taken	2	
	Daily	79	
	Once a week	14	
	Twice a week	5	
**Time preferred to send reminders**			
	Prior to expected ingestion events	2	
	Morning: 6 AM to 10 AM	80	
	Midday: 11 AM to 2 PM	11	
	Evening: 3 PM to 6 PM	1	
	Late evening or night: 7 PM to 10 PM	6	
	Any time	0	
**Would you use mobile phone to talk to your doctor or health worker?**			
	Yes, definitely	78	
	Yes, sometimes	18	
	Not sure	3	
	Very rarely	1	

^a^ATT: antitubercular therapy.

## Discussion

### Principal Findings

Over the past 10 years, there has been an exponential increase in ownership and usage of mobile phones in India with 1 billion wireless subscribers, expanding possibilities for mobile health–based interventions to address chronic disease [14,16]. Digital health technologies that leverage the use of mobile phones may therefore become widely accessible and used in the Indian context, given the mixed literacy rates and low income [16-18]. Among disease states, adherence to ATT is an attractive target for intervention using mobile phone technology [15,19]. Traditional directly observed treatment, short-course (DOTS) has had suboptimal uptake during the COVID-19 pandemic due to public health measures, and while relaxation of these measures may improve DOTs access, the use of digital health–mediated ATT adherence monitoring is promising [20]. Therefore, it is essential to identify, develop, and implement interventions that support adherence remotely. This study demonstrates a high uptake of mobile phones among patients with TB in Thrissur and their willingness to accept it as a method to provide mobile phone–based reminders such as voice calls to ensure treatment adherence to ATT. The results of this investigation also suggest that a structured intervention supporting ATT adherence using mobile devices in rural setting, such as that in our study, may need to use voice calls as their mainstay.

We found high uptake of mobile phones consistent with literature from India, which has reported up to 81% mobile phone uptake in remote areas of the country [15,21-23]. The study also confirms the high uptake of mobile phones in Thrissur in which the majority of the population is from a rural setting, suggesting the development of mobile phone–based ATT adherence supports could reach individuals on ATT. Importantly, most participants own their phone (84%), can operate the calling function (95%), and would be willing to interact with ATT adherence interventions on their phones (95%). Even individuals who leverage phones shared among multiple family members were willing to receive ATT adherence messages despite the fact that they may be received by another family member. Among these interventions, participants identified the ability to receive reminders and communication from health care providers as highly desirable. This suggests that future interventions could seek to leverage mobile phones as a platform to understand and reinforce ATT adherence.

Importantly, we discovered that most of the participants’ reminder preference is voice calls. Although daunting, the deployment of call center–based adherence reminders and monitoring may be a potential avenue through which population-level adherence interventions around ATT can be enacted. Although phone call–based adherence monitoring may be an indirect measure of adherence, it represents a universal platform that is accessible to those who may potentially most need adherence monitoring compared to other programs such as 99DOTS and smart pill bottles. Only a third of the participants reported competence with the SMS function, and 61% could use the camera function on their phones. ATT support via mobile phones as voice calls would be feasible and effective in Kerala. The SMS or camera or video-based interventions might require additional support for patients with TB in Kerala. For interventions that rely on SMS text messaging, instruction and support for SMS may need to be integrated into training modules prior to deployment. Voice-based interventions may be feasible in Kerala and eliminate the need for all individuals to travel to the DOTS clinic, thus eliminating this barrier to adherence. A potential system may deploy a call center–based ATT adherence system for individuals on ATT. In order to scale up, programs may consider prerecorded messages or potentially interventions grounded in a voice chat bot that delivers empiric ATT adherence interventions through voice calls. These systems should additionally consider downstream measures to address nonadherence or nonresponse to voice calls. For example, instead of using DOTS for all individuals on ATT, voice calls could potentially identify individuals who have the most difficulty with adherence, and in-person DOTS may be selected an alternative adherence measure.

In our survey, most of the participants were willing to receive adherence support through mobile phones, and they do not think that it invades their privacy. In addition, many of our participants do not fear the stigma of disclosure of their illness if a reminder about their treatment happens to be seen or received by others. These findings could be attributed to the success of support services offered to patients with TB, creating awareness about the disease among them [24]. Previous studies conducted in other rural areas of India have also reported similar responses [15,23]. However, investigations conducted among the general population found that the stigma surrounding TB still exists. Hence, continued awareness and support programs among patients with TB and the general population are necessary [25,26].

If given a choice, three-fourth of the study population would prefer to use mobile phone technology instead of conventional DOTS. Previous investigations have also shown that the users of 99DOTS reported a need for increased mobile phone support and potential willingness to use mobile phone reminders to improve adherence and ATT monitoring [15]. Based on our findings, enhancing the current 99DOTS program with more human interactions including direct phone calls or automated phone call reminders may be a possible avenue to overcome the pitfalls and strengthen the program.

### Limitations

Given that this study was a pilot study carried out only in a single city in South India, it may not be generalizable to the rest of the country. Similar study conducted in another state in South India shares similar results [15]. However, when India as a whole is considered, mobile phone uptake is only 40%; so, generalizability is a concern [27]. We only enrolled people coming to the TB treatment centers, thus resulting in missing nonadherent individuals whose responses could have been different. Further, as the study staff involved in administering the questionnaire were health care personnel, social desirability is likely to have influenced some of the responses, especially those focusing on acceptance of mobile phone–based ATT support.

## Appendix

Multimedia Appendix 1 Questionnaire tool used for the survey.

## Abbreviations

ATT: antitubercular therapy
DOTS: directly observed treatment, short-course
MDR-TB: multidrug-resistant tuberculosis
MEMS: medication event monitoring systems
TB: tuberculosis
TTC: Tuberculosis Treatment Center
vDOT: Video Directly Observed Therapy
