# Supplementary material for: The Acceptability of Adherence Support via Mobile Phones for Antituberculosis Treatment in South India: Exploratory Study
Source: JMIR Form Res. 2022 May 13;6(5):e37124. doi: 10.2196/37124 (PMC9143769; doi:10.2196/37124)
Supplement: Multimedia Appendix 1 [file formative_v6i5e37124_app1.doc]

**PID**: **Date of Visit (dd/mm/yyyy):**

| **Q1.** | **Name:** | **Q2.** | **Sex:** 1. Male 2. Female |
| --- | --- | --- | --- |
| **Q3.** | **Marital Status:** 1.Married 2.Unmarried 3.Widowed 4.Separated | **Q4.** | **Residence**:  Urban  Rural |
| **Q5.** | **Education:** 1. No formal 2. School education 3. Graduate 4. Post Graduate | **Q6.** | **Age:** |
| **Q7a.** | **Are you currently working?** 1. Yes 2. No  *If no, Goto Q7c.* | **b.** | **If yes:**  *Goto Q8* |
| **c.** | **What type of non-economic (non-work):** 1. Housewife 2. Unemployed 3. Other: | | |
| **Q8.** | **Language (mother-tongue):** 1. Malayalam 2. Tamil 3. Hindi 4. English 5. Other: | | |
| **Q9.** | **Read & Write:** 1. Malayalam 2. Tamil 3. Hindi 4. English 5. Other: | | |

**Clinical Details:**

| **Q10.** | **TB Diagnosis (dd/mm/yy):** | **Q11.** | **Start date of Treatment:** | | |
| --- | --- | --- | --- | --- | --- |
| **Q12.** | **Registration Group:** 1. New case 2. Relapse 3. Default 4. Failure | | | | |
| **Q13.** | **Type of TB:** 1. Pulmonary 2. Extrapulmonary, Specify: | | | | |
| **Q14.** | **Sputum Type:** 1. Positive 2. Negative | | | | |
| **Q15.** | **HIV Status:** 1. Positive 2. Negative 3. Unknown | | | | |
| **Q16.** | **Treatment phase:** 1. Intensive 2. Continuation  *If continuation* *phase*, Treatment supervisor: | | | | |
| **Q17.** | **Category of Treatment:** 1. I 2.II 3.DOTS Plus 4.Non DOTS  *If DOTS:* 1. Hospital based 2. Community based | | | | |
| **Q18.** | **a. Do you travel to reach your DOTS provider:**  *If No, Goto Q19* | | | Yes | No |
|  | **b. Distance to DOTS provider: ___________** *(mins)* | | | **c. Transport cost: Rs.** | |
| **Q19.** | **Have any of your DOTS appointments been missed?** (Either by you or councillor) *If yes, why?* | | | Yes | No |

**Cells highlighted in grey should be filled out from the patient’s chart*

| **Q No.** | **Phone Functionality** | **Responses** | | **Instructions** |
| --- | --- | --- | --- | --- |
| **Q20.** | Do you routinely use mobile phones? | Yes | No | If ‘Yes’ go to Q22 |
| **Q21.** | Why do you not use a mobile phone? (tick all that applies) | 1. Lack of money 2. No network 3. Have no use for it 4. Inability to use 5. Other: | | Document reason. More than one reason is possible. Do not prompt. |
| **Q22.** | Phone Ownership details:  *If share; with who?* | 1. Own phone 2. Own phone but share 3. Share phone 4. No phone | | If ‘No phone’ skip to Q33  Document who they share with. |
| **Q23.** | Since when have you used mobile phones? | _____yrs | | Fill in years (eg. 2, 2.5, 0.5 etc) |
| **Q24.** | Do you use a mobile phone to talk? | Yes | No | If no, skip to Q26 |
| **Q25.** | How often do you:   1. call others 2. receive calls | ___/day or wk  ___/day or wk | | Mark carefully whether day or week. |
| **Q26.** | Do you use the SMS function on your mobile phone? | Yes | No | If no, skip to Q28 |
| **Q27.** | How often do you   1. send SMS 2. receive SMS | ___/day or wk  ___/day or wk | | Mark carefully whether day or week. |
| **Q28.** | Do you use the alarm function? | Yes | No | If no, skip to Q30 |
| **Q29.** | What do you use the alarm function for? (tick all that applies) | 1. To wake up 2. To remind me of errands 3. Reminder for medicines 4. Reminder for DOTS visits 5. Other: | | Multiple answers possible. If other specify. |
| **Q30.** | Does your phone have a camera function? | Yes | No | Don’t know |
| **Q31.** | Do you know how to use the camera function? | Yes | No |  |
| **Q32.** | What other use do you have for the mobile phone? (tick all that applies) | 1. Listen to radio 2. Play games 3. Watch or stream video 4. Other: 5. None | | Multiple answers possible. If other specify. |
| **Q No.** | **Reminder Preference** | **Responses** | | **Instructions** |
| **Q33.** | If we were to provide you with interactive reminders to take medications, what format would you like these reminders to be in? | 1. Telephone call (Voice format) 2. SMS message 3. No preference 4. Both | | Choose only 1.  If pt chooses 2. then skip to Q35 |
| **Q34.** | Which language would you like the telephone call to be in? | 1. Malayalam 2. English 3. Other: | | If other specify. |
| **Q35.** | Which language would you like the SMS to be in? | 1.Malayalam  2.English  3.Other: | | If other specify. |
| **Q36.** | If we were to provide automatic reminders for medication in this way, how often would you like to receive them? | 1. As often as the medications need to be taken 2. Daily 3. Once a week 4. Twice a week | | Choose only 1. |
| **Q37.** | If automatic reminders were sent, what times would you like them sent? | 1. Just before the drugs timings in the morning and evening 2. Morning: 6am – 10 am 3. Mid day: 11 am – 2 pm 4. Evening: 3 pm – 6 pm 5. Late evening/night: 7 pm – 10 pm 6. Anytime | | Choose 1. |
| **Q38.** | Why is this time convenient for you? |  | |  |
| **Q39.** | If we were going to develop an application using mobile phones for TB patients in India– what other possibilities do you think would be useful? (tick all that applies) | 1. Communication with counsellor/ health provider 2. Information on medicines 3. Messages on advances in TB 4. Other: | | If other specify below. |
| **Q40.** | Do you think the cell phone used in this way will be an intrusion in a person’s life? | 1. Yes 2. No 3. Don’t know | |  |
| **Q41.** | If the reminder was received by someone other than yourself, would you fear the risk of stigma? | 1. Yes 2. No 3. Don’t know | |  |

| **Q42.** | Given a choice, would you prefer to come to the *(hospital/community centre/Non DOTS)* to receive your medications or would you prefer to receive mobile phone reminders and take your medications at home? | 1. Continue with current program 2. Receive mobile phone reminders 3. Neither 4. Both | | Based on answer category treatment – use the correct term. |
| --- | --- | --- | --- | --- |
| **Q43.** | Why do you prefer (insert answer to Q42)? |  | | |
| **Q44.** | Do you think it would be useful to receive interactive mobile phone reminders to help remember to take your medication instead of the current program? | Yes | No | If no, ask why and record below. |
| **Q45.** | If you had a mobile phone, would you use it to talk to your doctor or health worker? | 1. Yes, definitely 2. Yes, sometimes 3. Not sure 4. Very rarely 5. Probably not | | Please ask subject for the reason for choice & write in comments section below |
| **Q46.** | a. In the past, have you used a phone (mobile or landline) for any of the following? | 1. To call the doctor / health worker when you were unwell 2. To know availability of the doctor 3. To know availability of medicines 4. To purchase medicines 5. Other: 6. Have not used phone to call for any reason | | If other specify below. |
|  | b. If yes, can you comment on how this has helped you? |  | |  |
